# Connecting epigenetics and inflammation in vascular senescence: state of the art, biomarkers and senotherapeutics

**DOI:** 10.3389/fgene.2024.1345459

**Published:** 2024-02-26

**Authors:** Oscar Fraile-Martinez, Diego De Leon-Oliva, Diego Liviu Boaru, Patricia De Castro-Martinez, Cielo Garcia-Montero, Silvestra Barrena-Blázquez, Joaquin García-García, Natalio García-Honduvilla, Melchor Alvarez-Mon, Laura Lopez-Gonzalez, Raul Diaz-Pedrero, Luis G. Guijarro, Miguel A. Ortega

**Affiliations:** ^1^ Department of Medicine and Medical Specialities, Faculty of Medicine and Health Sciences, University of Alcalá, Alcala deHenares, Spain; ^2^ Ramón y Cajal Institute of Sanitary Research (IRYCIS), Madrid, Spain; ^3^ Department of Surgery, Medical and Social Sciences, Faculty of Medicine and Health Sciences, University of Alcalá, Alcala deHenares, Spain; ^4^ Network Biomedical Research Center for Liver and Digestive Diseases (CIBEREHD), Madrid, Spain; ^5^ Immune System Diseases-Rheumatology, Oncology Service an Internal Medicine (CIBEREHD), University Hospital Príncipe de Asturias, Alcala deHenares, Spain; ^6^ Department of General and Digestive Surgery, General and Digestive Surgery, Príncipe de Asturias Universitary Hospital, Alcala deHenares, Spain; ^7^ Unit of Biochemistry and Molecular Biology, Department of System Biology (CIBEREHD), University of Alcalá, Alcala deHenares, Spain; ^8^ Cancer Registry and Pathology Department, Principe de Asturias University Hospital, Alcala deHenares, Spain

**Keywords:** cellular senescence, epigenetic modifications, immune activation, endothelial cells (ECs), vascular smooth muscle cells (VSMCs)

## Abstract

Vascular diseases pose major health challenges, and understanding their underlying molecular mechanisms is essential to advance therapeutic interventions. Cellular senescence, a hallmark of aging, is a cellular state characterized by cell-cycle arrest, a senescence-associated secretory phenotype macromolecular damage, and metabolic dysregulation. Vascular senescence has been demonstrated to play a key role in different vascular diseases, such as atherosclerosis, peripheral arterial disease, hypertension, stroke, diabetes, chronic venous disease, and venous ulcers. Even though cellular senescence was first described in 1961, significant gaps persist in comprehending the epigenetic mechanisms driving vascular senescence and its subsequent inflammatory response. Through a comprehensive analysis, we aim to elucidate these knowledge gaps by exploring the network of epigenetic alterations that contribute to vascular senescence. In addition, we describe the consequent inflammatory cascades triggered by these epigenetic modifications. Finally, we explore translational applications involving biomarkers of vascular senescence and the emerging field of senotherapy targeting this biological process.

## 1 Introduction to cellular senescence

By definition, cellular senescence is the state of the cell induced by stress signals characterized by cell-cycle arrest, senescence-associated secretory phenotype (SASP), metabolic dysregulation, and macromolecular damage (Gorgoulis et al., 2019). Cellular senescence was first formally described in 1961 by Hayflick and Moorhead (Hayflick and Moorhead, 1961). They observed that cultured human diploid fibroblasts exhibited a finite number of divisions (40–60 population doublings), a phenomenon attributed to intrinsic factors, which were later identified as telomere shortening. Different external and internal stress and developmental signals trigger cellular senescence in response to cellular damage, including telomere shortening, DNA damage, oncogenic activation, radiation, oxidative and genotoxic stress, epigenetic changes, perturbed proteostasis, mitochondrial dysfunction, inflammation, nutrient deprivation and mechanical stress (Kuilman et al., 2010; Muñoz-Espín et al., 2013; Storer et al., 2013; Hernandez-Segura et al., 2018; Kumari and Jat, 2021; Huang et al., 2022; Zhou et al., 2023).

On the one hand, cellular senescence displays diverse physiological roles, mainly in embryonic development, wound healing, and prevention of tumor development (Muñoz-Espín et al., 2013; Rhinn et al., 2019; Wilkinson and Hardman, 2020; Yang et al., 2021). On the other hand, cellular senescence is a hallmark of aging due to the accumulation of senescent cells in old tissues compared to young tissues (Tuttle et al., 2020; López-Otín et al., 2023). Senescent cells signal to be removed by immune cells from the tissues, triggering both innate and adaptive responses (Kale et al., 2020). However, with age, the cell-intrinsic damage and the failure of immune clearance increase the number of senescent cells in the tissues and contribute to the development and progression of aging-related diseases such as cancer, neurodegenerative diseases, atherosclerosis, osteoarthritis or pulmonary fibrosis (Burton and Stolzing, 2018; Ovadya et al., 2018; Mylonas and O’Loghlen, 2022).

Senescence is characterized by a cell-cycle arrest that can occur in the G1 or G2 phase (Gire and Dulic, 2015). The cell-cycle withdrawal is generally irreversible, although under certain circumstances senescent cells can re-entry the cell-cycle, such as tumor cells, or be reprogrammed into induced pluripotent stem cells (Lapasset et al., 2011; Saleh et al., 2019). Senescence differs from quiescent and terminally differentiated cells, which also withdraw from the cell-cycle, at the level of signaling pathways, and SASP and neither shows macromolecular damage (Terzi et al., 2016; Fujimaki et al., 2019). The cell-cycle arrest is achieved through different tumor suppressor pathways, especially p53/p21^WAF1/CIP1^ and p16^INK4A^/pRB tumor suppressor pathways (Kumari and Jat, 2021). p53 acts as a transcription factor (TF), which is activated in response to DNA damage caused by factors like telomere attrition, oxidative stress, or oncogenic stress, and orchestrates a genetic response that leads to the induction of cellular senescence and inhibits other alternatives such as apoptosis (Childs et al., 2014; Sheekey and Narita, 2023). Of the downstream effectors, the key role is predominantly played by p21^CIP1^, which is an inhibitor of cyclin-dependent kinase (CDK)-cyclin complex activity leading to cell-cycle withdrawal (Herranz and Gil, 2018). On the other hand, activation of p16 through epigenetic regulation leads to the inhibition of the formation of cyclin D–CDK4/6 complexes, which prevents the phosphorylation of the retinoblastoma protein (pRb) (Rayess et al., 2012). Hypophosphorylated pRb maintains in the cytoplasm in complex with the TF E2F and cell-cycle genes are not transcribed. Moreover, the two pathways present an extensive crosstalk. The hypothesis is that while the p53/p21^WAF1/CIP1^ signaling pathway contributes to the onset of cellular senescence, the p16^INK4A^/pRB pathway is in charge of maintaining this state (Kohli et al., 2021).

Almost all somatic cell types and tissues can undergo cellular senescence. The evidence of cellular senescence in the vasculature is termed vascular senescence and plays a critical role in the development of vascular diseases, including atherosclerosis, peripheral arterial disease, hypertension, stroke, diabetes, chronic venous disease and venous ulcers (Yamazaki et al., 2016; Katsuumi et al., 2018; Shakeri et al., 2018; McCarthy et al., 2019; Ortega et al., 2021b; Lim et al., 2021).

Epigenetics regulates cellular senescence through chromatin remodeling, DNA methylation, and the involvement of non-coding RNAs (Sidler et al., 2017; Crouch et al., 2022). Senescent cells undergo chromatin restructuring, altering DNA packaging around histones, which impacts gene accessibility through modifications such as methylation and acetylation (Roger et al., 2021). DNA methylation contributes to establishing and maintaining the senescent state by silencing specific genes related to cell proliferation (Sakaki et al., 2017). In addition, non-coding RNAs, such as microRNAs and long non-coding RNAs, modulate fundamental pathways in senescence by targeting genes related to cellular aging, DNA repair, and the cell cycle (Puvvula, 2019). Together, these epigenetic modifications coordinate the transition to cellular senescence by regulating genes related to cell cycle arrest, DNA damage response, SASP, and inflammation, thus shaping the phenotype of senescent cells.

Inflammation is a protective mechanism, essential for the immune system to detect and eliminate harmful agents while initiating the healing process. Inflammatory responses can manifest as acute or chronic, each serving different purposes (Li et al., 2023). However, not all inflammatory processes benefit the body; in some cases, diseases trigger harmful inflammation, in which the immune system inadvertently attacks the body’s cells. In this sense, senescent cells often release several inflammatory substances, such as matrix metalloproteinases (MMPs), growth factors (GFs), and cytokines (CKs), which form the SASP (Giacconi et al., 2015; Lee et al., 2021). This paracrine signaling contributes to a variety of adverse outcomes, such as cancer development, persistent inflammation called “inflammaging” and tissue restructuring (Olivieri et al., 2018). Moreover, senescent cells are indeed a strong nexus between cellular aging and development of cancer (Campisi, 2013; Schmitt et al., 2022; López-Otín et al., 2023). Pharmacological removal of senescent cells expressing p16^INK4A^ in aging mice delayed tumorigenesis and mitigated age-related decline in multiple organs, showing no apparent adverse effects (Baker et al., 2016).

In this comprehensive review, we aim to understand the interplay between epigenetic modifications and immune activation mechanisms underlying vascular senescence. First, we present an overview of the histology of the vascular wall and vascular senescence. Then, we explore the epigenetic alterations identified in the context of vascular senescence and elucidate the critical role played by immune activation. Subsequently, we sought to synthesize the connections between epigenetic modifications, the immune response, and the phenomenon of vascular senescence. Finally, we describe the translational implications arising from the fields of epigenetics, inflammation, and cellular senescence in the context of vascular disorders, including biomarkers and senotherapeutics.

## 2 Histology of vascular wall and vascular senescence

**FIGURE 1:**
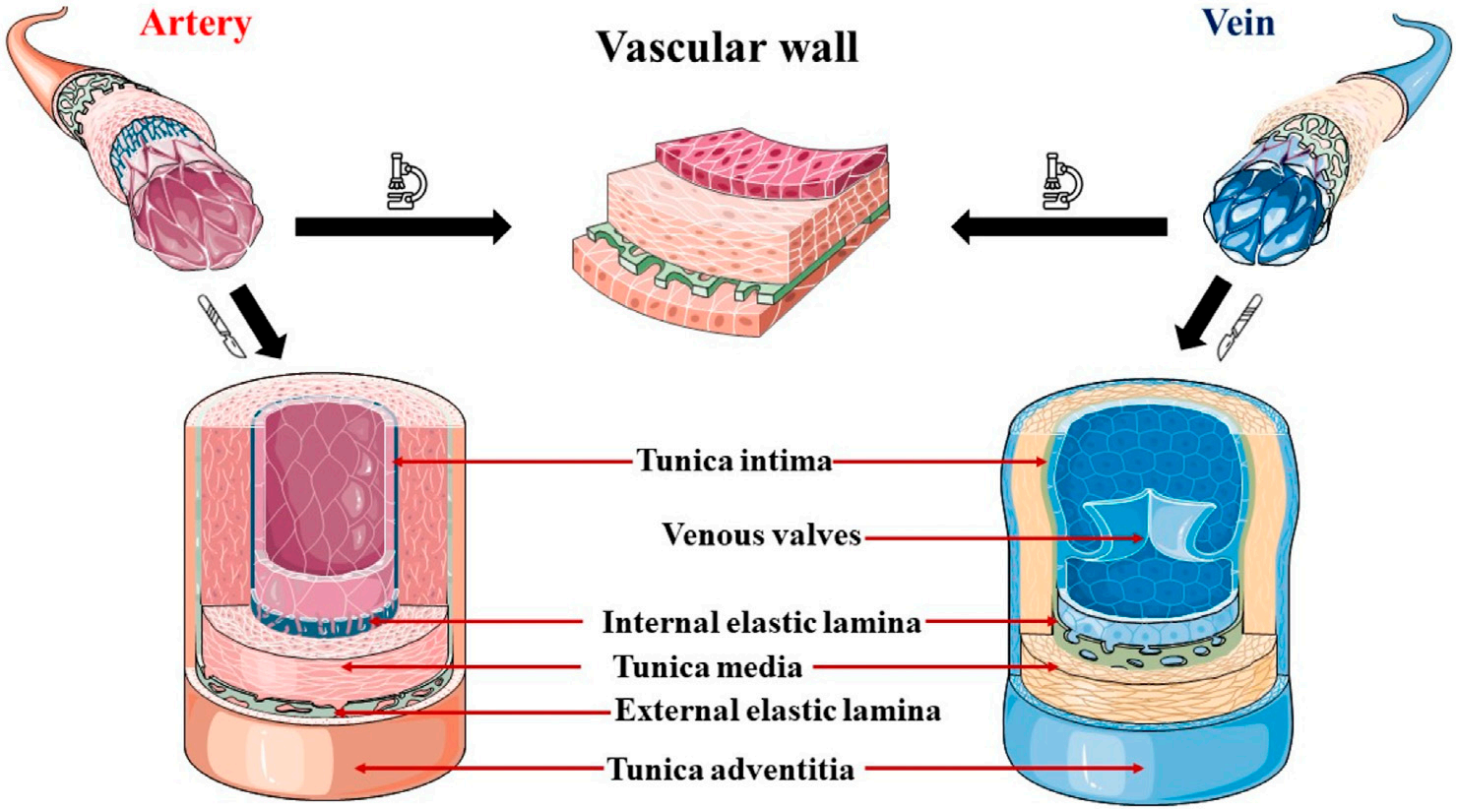
Representation of the histological structure of the vascular wall of arteries and veins. Arteries present a relatively thick tunica media and more VSMCs, whereas veins show a larger tunica adventitia. The internal elastic lamina appears in arteries and only in the median veins, where is thinner and discontinuous. The external elastic lamina of arteries is a layer composed of elastin that separates the tunica media and the adventitia. Finally, venous valves are folds of the tunica intima composed of dense connective tissue present in the middle veins, especially abundant in the lower extremities, to prevent the backflow of blood.

The vascular wall, a complex structure comprising three distinct layers, the intima, media, and adventitia, exhibits diverse histological, biochemical, and functional characteristics essential for maintaining vascular homeostasis and modulating the vascular response to stress or injury. From a histological point of view, the differences in thickness and composition of them distinguish the different types of blood vessels. In general, the tunica intima is formed by a single layer of endothelial cells (ECs) or endothelium, which surrounds the internal vascular lumen acting as a functional barrier (Taylor and Bordoni, 2023). The endothelium is supported by a basement membrane composed of collagen, proteoglycans, and glycoproteins (Halper, 2018). The subendothelial layer is composed of loose connective tissue, and sometimes harbors vascular smooth muscle cells (VSMCs), and an internal elastic lamina in arteries and some veins (Mazurek et al., 2017). The tunica media is stabilized by an elastic external lamina, in arteries, and an extracellular matrix composed mainly of elastin and collagen fibers (Munjal and Bordoni, 2023). The tunica media houses predominantly VSMCs arranged in circumferentially organized layers with elastin, reticular fibers, and proteoglycans between them (Frismantiene et al., 2018). The outermost layer, the adventitia, has myofibroblasts and fibroblasts as the main cellular constituents, responsible for generating the fibroelastic extracellular matrix that defines the appearance of this layer (Stenmark et al., 2013). In addition, in larger vessels, the adventitia harbors a network of small blood vessels and nerves, which provide irrigation and innervation to the vascular wall, respectively. Figure 1 illustrates the histological representation of these layers in both arteries and veins, as well as their main differences.

**FIGURE 2:**
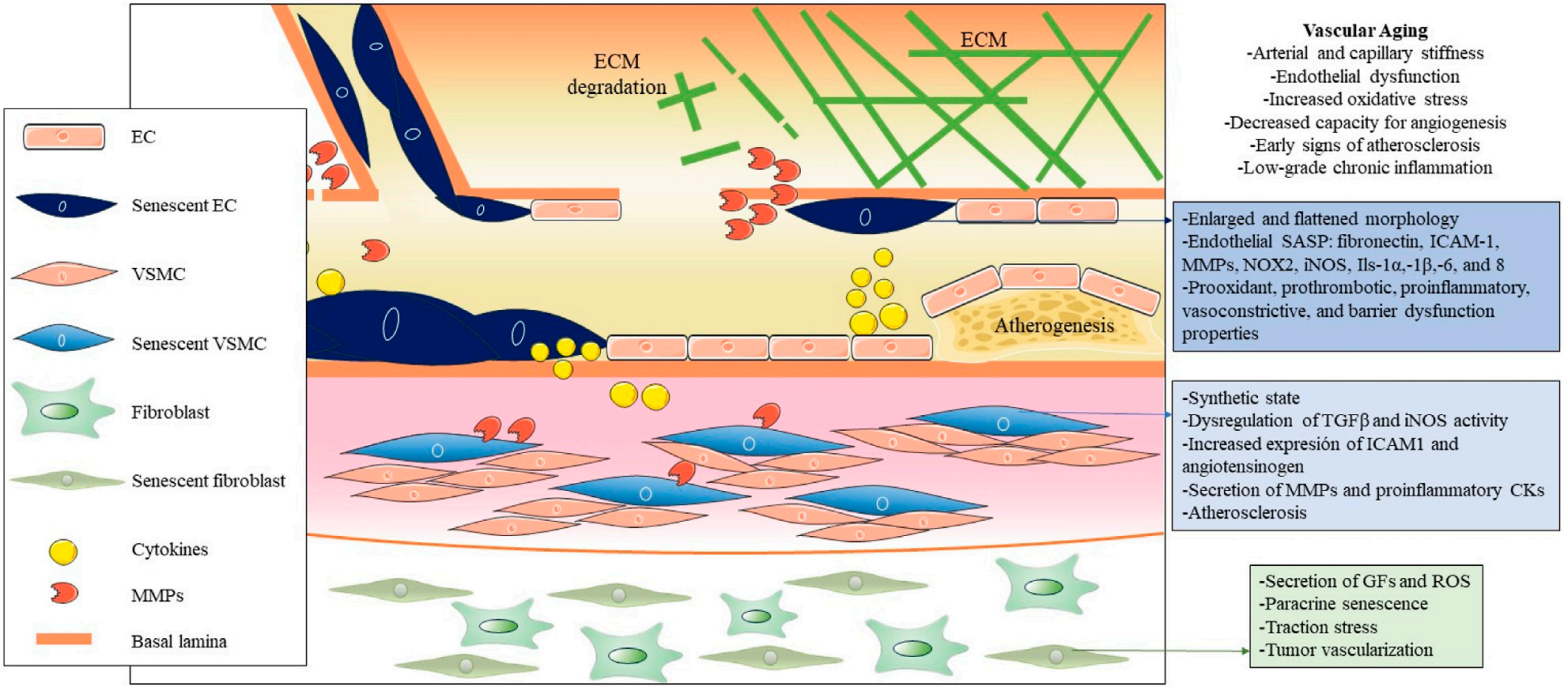
Pathogenic influence of cellular senescence in vascular aging. The figure illustrates the pathogenic impact of cellular senescence in vascular aging, depicting the main cellular and molecular entities associated with senescence in this context. Throughout the three main layers that compose the vascular wall, distinct senescent cell types, predominantly endothelial cells (ECs), vascular smooth muscle cells (VSMCs), and fibroblasts, are distinguished from the inside out. Due to their decreased functionality and secretion of SASP factors, these cells collectively contribute to vascular dysfunction, altered permeability, increased inflammation and oxidative stress, and the onset of atherogenesis in arteries.

Cellular senescence in blood vessels plays a critical role in vascular aging, and the elimination of senescent cells is a promising approach to prevent or mitigate age-related vascular diseases (Figure 2) (Ungvari et al., 2020). Manifestations of vascular aging include arterial and capillary stiffness, endothelial dysfunction, increased oxidative stress, decreased capacity for angiogenesis, early signs of atherosclerosis, and low-grade chronic inflammation (Ghebre et al., 2016). Vascular aging constitutes a progressive decline in both the structural integrity and functional capacity of blood vessels, which contributes to damage to the heart, brain, kidneys, and other organs (Climie et al., 2023; Ya and Bayraktutan, 2023). It is important that, while there is extensive literature on arterial aging and related diseases, there is a significant knowledge gap on venous aging. However, research on the role of venous aging is of great importance in the understanding of the development of venous conditions prevalent among older adults, such as varicose veins, chronic venous insufficiency, and deep vein thrombosis (Ortega et al., 2021a; Ortega et al., 2021b; Molnár et al., 2021; Mühlberger et al., 2022).

The main cellular populations of the blood vessels that can undergo cellular senescence due to various stressors like oxidative stress, DNA damage, and signaling from nearby cells are ECs, VSMCs, adventitial fibroblasts, immune cells and endothelial progenitor cells (EPCs) (Katsuumi et al., 2018; Vellasamy et al., 2022; Suda et al., 2023).

### 2.1 Endothelial cell senescence

EC senescence is a key feature of the aging process in blood vessels. These cells, which are vital for vascular homeostasis, undergo senescence during aging or due to stimuli such as reactive oxygen species (ROS), chronic inflammation, altered blood flow patterns, metabolic influences such as glucose, insulin and specific lipid subfractions (Khan et al., 2017; Hwang et al., 2022), while molecules like polyphenols, amino acids, and omega-3 fatty acids display senescence-inhibiting effects (Sakai et al., 2017; Furuuchi et al., 2018; Tsuboi et al., 2018). This transition confers prooxidant, prothrombotic, proinflammatory, vasoconstrictive, and barrier dysfunction properties on ECs, altering their crucial role in maintaining healthy blood flow and vessel integrity (Ramírez et al., 2022; Han and Kim, 2023). Senescent ECs show the typical enlarged and flattened morphology of senescence. Senescence in ECs shows changes in molecular profiles and contributes to the distinctive endothelial SASP, including higher expression of fibronectin, intercellular adhesion molecule 1 (ICAM-1), matrix metalloproteinases (MMPs), NADPH oxidase 2 (NOX2), inducible nitric oxide synthase (iNOS), interleukins 1α 1β, 6, and 8, and decreased production of nitric oxide (NO) (Hayashi et al., 2006; Zhou et al., 2006; Yin and Pickering, 2016; Rojas et al., 2017). Interestingly, exercise counteracts EC senescence by modulating oxidative stress and inflammatory pathways, preserving vascular function during aging, and offering a promising avenue to counteract age-related vascular complications (Rossman et al., 2017; Meng et al., 2023). Also, circulating endothelial progenitor cells in patients with coronary artery disease exhibited reduced telomere length and telomerase activity *via* oxidative DNA damage, which may be related to EC senescence (Satoh et al., 2008).

### 2.2 Vascular smooth muscle cells senescence

Aged blood vessels show decreased compliance, elasticity, and distensibility, as well as increased stiffness (Kohn et al., 2015). These alterations contribute to higher systolic blood pressure and lower diastolic blood pressure. This is explained by the accumulation of collagen and decay of elastin in the layers of the arterial wall (Mitchell, 2021).

However, senescent VSMCs switch from a contractile to a synthetic state. This alteration involves dysregulated transforming growth factor-β (TGF-β) signaling, increased iNOS activity, and increased expression of ICAM-1 and angiotensinogen under stress conditions (Gorgoulis et al., 2005; Lakatta, 2015; You et al., 2019). Particularly, senescent VSMCs tend to accumulate in atherosclerotic plaques, contributing to inflammation, extension and vulnerability of the plaque by different mechanisms: reduced collagen production, secretion of MMPs, proinflammatory cytokines (IL-1α,-6 and,-8 and MCP1), chemotaxis of monocytes/macrophages, acquisition of an “osteoblast-like” phenotype (Nakano-Kurimoto et al., 2009; Gardner et al., 2015; Lacolley et al., 2017). Recent findings indicate that senescent VSMCs in atherosclerotic plaque have decreased levels of telomeric repeat-binding factor-2 (TRF2), a protein crucial for telomere protection (Wang et al., 2015; Uryga et al., 2021). *In vivo* studies with transgenic mice confirm that inhibition of VSMC senescence, by modulating TRF2, has the potential to prevent atherosclerotic disease progression.

### 2.3 Fibroblasts senescence

Senescent vascular fibroblasts, found in the adventitia of blood vessels, secrete proinflammatory molecules and contribute to vascular remodeling. Their secretion of bioactive substances, such as GFs and ROS, creates a pro-oxidant environment that may favor age-related vascular pathologies, such as pulmonary hypertension (Li et al., 2021). Furthermore, it has been demonstrated in fibroblasts that senescent cells can influence intact neighboring cells through a bystander effect, which induces a DNA damage response, propagating senescence in the vascular microenvironment (Nelson et al., 2012). Additionally, it was demonstrated in A 3D tissue model-on-a-chip, that senescent fibroblasts also exert excessive traction stress on the surrounding extracellular matrix (ECM) (Pauty et al., 2021). In particular, senescent fibroblasts also promote tumor vascularization by inducing increased expression of vascular endothelial growth factor (VEGF), showing a relevant influence on the tumor microenvironment (Coppe et al., 2006).

### 2.4 Immune cells senescence

**FIGURE 3:**
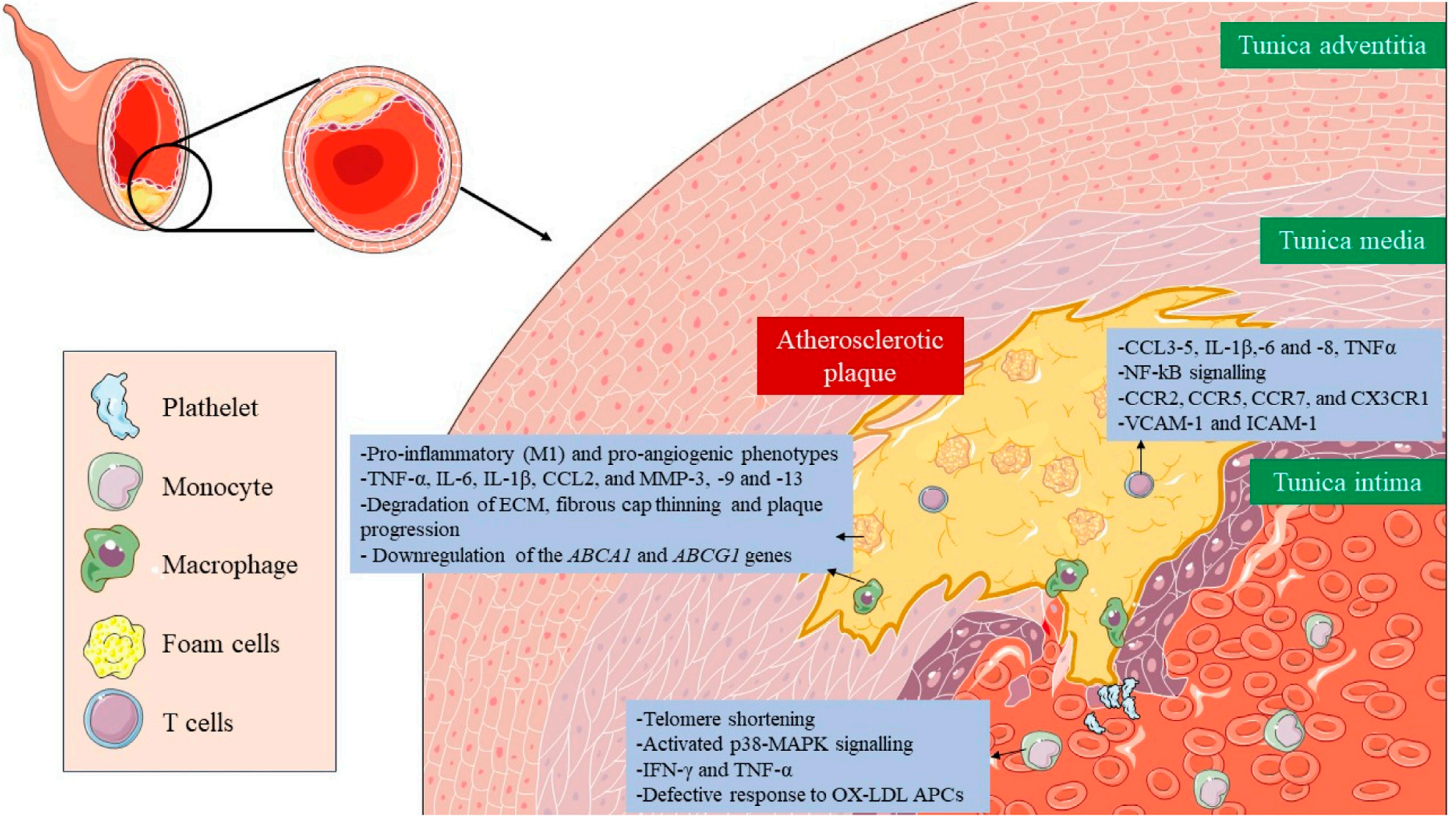
Role of senescent immune cells in atherosclerotic plaque. The senescence of immune cells, including monocytes, macrophages, foam cells, and T lymphocytes, plays a critical role in the formation, development and rupture of atherosclerotic plaque. Altered cellular functionality due to immunosenescence results in the secretion of proinflammatory cytokines, amplifying plaque inflammation and facilitating the recruitment of additional immune cells. Taken together, immunosenescence has a major impact on plaque stability, interactions, and potential therapeutic approaches targeting these senescent immune components.

Immunosenescence is a phenomenon in which the immune system gradually declines with age (Wang Y. et al., 2022). The senescence of immune cells plays a pivotal role in the development of atherosclerosis (Figure 3) (Vellasamy et al., 2022). The cellular senescence of monocytes and macrophages changes their functionality, contributing to chronic inflammation and pathological processes associated with atherosclerosis, which ultimately impact plaque development and stability and the risk of cardiovascular events. All three monocyte subsets, classical (CD14++CD16^−^), intermediate (CD14++C, D16+), and non-classical (CD14^+^CD16++), show characteristics of senescence, particularly the nonclassical subset, which increases with age (Ong et al., 2018). This subset had markedly elevated levels of TNF-α, CCL3, and CCL4, whereas IL-6, IL-8, IL-1β, and CCL5 were secreted at comparatively high levels in both the intermediate and non-classical subsets. This subset also expresses membrane-bound IL-1α and exhibits increased NF-kB signaling (Zawada et al., 2011). Senescent monocytes, especially the non-classical subset, express proatherogenic chemokine receptors (CCR2, CCR5, CCR7, and CX3CR1) and endothelial adhesion molecules (VCAM-1 and ICAM-1), which increases their adherence to vascular walls and contributes to the development of atherosclerotic plaque (Merino et al., 2011).

In the case of senescent macrophages, they adopt pro-inflammatory (M1) and pro-angiogenic phenotypes characterized by the secretion of SASP factors, such as TNF-α, IL-6, IL-1β, CCL2, and MMP-3, -9 and -13, which also lead to degradation of ECM, fibrous cap thinning, plaque progression, and instability (Sun et al., 2022; Zhu et al., 2023). They also present a disruption in cholesterol efflux due to downregulation of the *ABCA1* and *ABCG1* genes (Sene et al., 2013; Lin et al., 2018). Lastly, senescent foam cells accumulate in the subendothelial space and work in a very similar way to macrophages contributing to atherosclerosis (Childs et al., 2016).

Lastly, senescence in T cells presents implications for vascular disorders. Senescence is induced after oxidative stress leads to a reduction in telomerase activity, and consequently, telomere shortening, and the acquisition of a proinflammatory phenotype, with the secretion of IFN-γ and TNF-α (Mittelbrunn and Kroemer, 2021; Shirakawa and Sano, 2021). They have activated the p38 mitogen-activated protein kinase (p38-MAPK) signaling pathway (Reustle and Torzewski, 2018). Different subsets of senescent T cells appear also within the atherosclerotic plaque, such as CD4+/CD8+ TEMRA, CD8+/CD4+CD28^−^ or CD8^+^CD57^+^CD27^−^CD28^null^ T cells, where they promote inflammation and present a defective response to oxidized low-density lipoproteins (oxLDL) antigen presenting cells (APCs) (Kaplan et al., 2011; Yu et al., 2016).

## 3 Epigenetic modifications and vascular senescence

Epigenetic mechanisms are fundamental drivers of cellular senescence (Nacarelli et al., 2017; Crouch et al., 2022). Expression of the “Yamanaka factors” demonstrates their potential to restore cellular pluripotency by effectively eliminating epigenetic memory in differentiated cells through loss of heterochromatin and reducing the levels of repressive histones H3K9me2, H3K9me3, and 5-methylcytosine (Takahashi and Yamanaka, 2006; Wang K. et al., 2022). Furthermore, epigenetic alterations are also a hallmark of aging (López-Otín et al., 2023). Therefore, it is of great interest to study their connection with cellular senescence, with a special focus on vascular senescence in this review. The epigenetic alterations include DNA methylation, histone modifications, non-coding RNA-based gene regulation, and remodeling of chromatin landscape, and are correlated with cardiovascular risk factors and vascular aging (Ding et al., 2018; Zhang et al., 2018; Lin et al., 2022). To deepen our understanding of the importance of epigenetic regulation, cellular senescence can be induced *in vitro* by epigenetic modifiers, which activate the corresponding molecular pathways, based on the upregulation of p16^INK4A^ (Petrova et al., 2016). These include DNA methyltransferases inhibitors (5-aza-2- deoxycytidine), histone deacetylases inhibitors (sodium butyrate, trichostatin A), histone acetyltransferases inhibitors (curcumin, C646) or histone methyltransferases inhibitors (e.g., BRD4770).

DNA methylation is an important regulator of gene expression in vascular senescence (Ding et al., 2020; Xu et al., 2021). Environmental signals influence the activity of DNA methyltransferases (DNMT1) 1, 3a, and 3b (Denis et al., 2011). For instance, the deficiency of folic acid, a DNMT inhibitor, is correlated with an increased risk of cardiovascular disease (CVD), including coronary heart disease, atherosclerosis, and anemia (Glier et al., 2014; Voelter-Mahlknecht, 2016). The folic acid supplementation delays atherosclerotic lesion development by modulating *MCP1* and *VEGF* DNA methylation levels (Cui et al., 2017). DNMT1 has been identified as an essential factor in the formation of senescence-associated heterochromatin foci (SAHF) through the upregulation of HMGA2 (Sati et al., 2020). SAHF are domains of facultative heterochromatin in senescent cells that repress the expression of genes related to proliferation and are another biomarker of cellular senescence (Aird and Zhang, 2013). Abnormal DNA methylation patterns, prevalent in aging cells, have been widely associated with various age-related vascular diseases, hence their importance in vascular senescence (Tabaei and Tabaee, 2019; Xu et al., 2021).

Histone acetylation stands as a critical epigenetic regulator in vascular senescence, modulating chromatin accessibility and gene expression. The regulation of histone acetylation involves histone deacetylases (HDACs) and histone acetyltransferases (HATs). Regarding EC senescence, HDAC3 activated by laminar flow and VEGF through the VEGF receptor 2/protein kinase B pathway, stimulates EPC proliferation and EC differentiation (Zeng et al., 2006). In contrast, suppression of HDAC3 disrupts VEGF-induced EPC function. Furthermore, shear stress increases HAT activity, driving differentiation of the mouse embryonic stem cells into EC lineage (Illi et al., 2005). On the other hand, the interaction of Sirt1 with the PAI-1 promoter inhibits the acetylation of lysine 16 of histone H4, exerting a protective effect against vascular endothelial cell senescence (Wan et al., 2014). Also, Ang II induced protein kinase B-mediated phosphorylation and lysine acetylation of PGC-1 through the general control histone acetyltransferase nonderepressible 5, resulting in reduced PGC-1 activity and catalase expression in vascular cells (Xiong et al., 2010). Different studies have highlighted specific histone methylation markers, such as H4K20me3, H3K9me3, and H3K27me3, associated with the aging process (Yi and Kim, 2020). Decreased expression of H3K27me3 has been observed in aged hematopoietic stem cells (HSCs), whereas decreased expression of H3K9me3 has been observed in mesenchymal stem cells (MSCs) from aged individuals (Cakouros and Gronthos, 2019; Wang K. et al., 2022).

Non-coding RNAs, microRNAs (miRNAs), and long non-coding RNAs (lncRNAs, play important regulatory roles in vascular senescence. The miRNAs, approximately 25 nucleotides in length, play a key role in regulating EC senescence and vascular aging (Lee et al., 2015; Lin et al., 2016; Nikolajevic et al., 2022). Several miRNAs are involved in processes such as vascular growth, angiogenesis, inflammation, and fibrosis (Table 1). In addition, miRNAs may play a role in senescence distinct from their traditional functions. For example, Argonaute 2 (AGO2) forms a complex with pRB in the nucleus by binding to let-7f, resulting in restrictive chromatin at the CD2 and CDCA8 promoters (Benhamed et al., 2012). lncRNAs also influence vascular diseases by regulating ECs and VSMCs. Loss of lncRNA H19 increases p21 and p16 expression, leading to EC senescence (Hofmann et al., 2019). LncRNA MEG3, elevated in the aged atrium and human umbilical vein endothelial cells (HUVECs), suppresses the proliferation of ECs through interaction with miR-9 and has a regulatory role on angiogenesis through the notch signaling pathway (Jayasuriya et al., 2022). Exosomal lncRNA GAS5 is shown to regulate the apoptosis of macrophages and ECs in atherosclerotic plaques (Chen et al., 2017).

**TABLE 1:** Role of different miRNAs with impact on vascular senescence.

miRNAs	Target genes	Role in vascular senescence	References
miR-504	*p53*	Lowers p53 activity and abundance and promotes VSMC dysfunction	Reddy et al. (2016)
miR-122	*CPEB*	Reduces translation and polyadenylation of p53 mRNA in fibroblasts	Burns et al. (2011)
miR-605	*MDM2*	Activates p53-mediated senescence and downregulates p21^WAF1/Cip1^ in HMECs and regulates secretion of CXCL5 in ECs	Borgdorff et al. (2010), Pande et al. (2021)
miR-17–3p	*Par4*	Negative modulator of cardiac aging and cardiac fibroblast cellular senescence	Du et al. (2015)
miR-126–5p	*Dlk1*	Promotion of the proliferation of ECs	Schober et al. (2014)
miR-21	*PTEN*	Abnormal proliferation of VSCMs	Hutcheson et al. (2014)
miR-206	*VEGF*	Regulation of CAD progression	Wang et al. (2016a)
miR-23a	*EGFR*	Inhibition of cell migration and vasculogenesis of CAD	Wang et al. (2016b)
miR-361–5p	*VEGF*	Antiangiogenic effect in acute coronary syndrome	Wang et al. (2014)
miR-574–5p	*ZDHHC14*	Proliferation of VSMCs and inhibition of apoptosis in CAD	Lai et al. (2018)
miR-33	*ABCA1/ABCG1*	Inhibition of cholesterol efflux in aged macrophages	Ono (2016)

Nuclear lamins are structural proteins of the nuclear envelope and are classified as V-type intermediate filaments. These lamins are divided into two main types based on their isoelectric points: A-type (which includes lamins A and C) and B-type (B1 and B2). Among these, lamin B1 is of particular importance in ensuring organogenesis and survival of the organism (Kim et al., 2011). Research indicates that cellular senescence, observed in both human and mouse cells, leads to a depletion of lamin B1 (Freund et al., 2012). Interestingly, this depletion occurs due to direct stimulation of the p53 or pRB pathway, independent of several typical cellular markers of senescence, such as p38, NF-κB, DNA damage response, or ROS. In contrast to apoptosis, in which caspases cleave lamins, senescent cells do not show lamin B1 cleavage products. Furthermore, inhibition of caspases does not affect the loss of lamin B1 during senescence (Shah et al., 2013). Instead, decreased stability of lamin B1 mRNA contributes to reduced levels of lamin B1 mRNA during the senescence process. Thus, lamin B1 depletion emerges as a robust indicator of cellular senescence, applicable both *in vitro* and *in vivo*.

## 4 Connecting inflammation with vascular senescence

A significant aspect of the changes in gene expression associated with aging is the increased activation of genes related to inflammation and immune responses. Indeed, chronic inflammation is another hallmark of aging and increases with age, known as inflammaging (Li et al., 2023). This phenomenon is related to the interaction between inflammation and cellular senescence, which favors the pathogenesis of numerous age-related vascular diseases (Del Pinto and Ferri, 2018; Liberale et al., 2022). This interaction is bidirectional, as inflammation spreads cellular senescence and senescent cells promote inflammation by secreting SASP factors (Stojanovic et al., 2020; Zhu et al., 2021). In addition, the function of the immune system decreases with age, termed immunosenescence, and thus the elimination of senescent cells, which promotes senescence-induced inflammation. The relationship between inflammation and vascular senescence can be explained by the involvement of specific biomarkers and signaling pathways.

IL-6 is emerging as a member of the interleukin family showing promise as a biomarker for aging and a reliable indicator of low-grade inflammation (Puzianowska-Kuźnicka et al., 2016). It is correlated with markers of aging such as carotid intima-media thickness (cIMT) and plaque progression (Huang et al., 2016). Moreover, IL-6 has been implicated in hypertension as its levels decrease with angiotensin II receptor blockade therapy suggesting a link to blood pressure in hypertensive patients (Stevenson et al., 2018). C-reactive protein (CRP), an acute phase reactant regulated by IL-6 and IL-1, does not act as a general marker of inflammation but also poses a significant risk factor for age-related conditions. Elevated CRP levels have been associated with declines in physical abilities. They are correlated with conditions such as CVD, hypertension, diabetes mellitus, and kidney disease (Tang et al., 2017). GDF 15, a member of the superfamily transforming growth factor β (TGF β), stands out particularly due to its controversial association with age and EC senescence. On the one hand, GDF15 can promote EC senescence through a p16 ROS-mediated pathway and contribute to atherosclerosis through pro-senescent activity (Park et al., 2016). On the other hand, the paracrine effects of GDF15 in non-senescent ECs showed that GDF15 increased proliferation, migration, and NO production and activated several signaling pathways such as AKT, ERK1/,2, and SMAD2 without triggering any oxidative stress, and therefore, preventing the endothelial dysfunction (Ha et al., 2019).

The SASP comprises a set of various substances released by senescent cells, which play a key role in maintaining tissue balance or precipitating dysfunction. This phenomenon is one of the main ones responsible for the adverse effects associated with senescent cells. Within the SASP, these cells release an array of substances, such as growth factors, metalloproteinases, cytokines, chemokines, and extracellular vesicles (EVs), which profoundly influence immune signaling and cell-cell communication (Ohtani, 2022; Sun et al., 2022). We have reviewed, i.e., section 2 the main SASP factors secreted by senescent cells affecting the vascular wall, which contribute to inflammation, and oxidative stress and lead to vascular dysfunction and disease. Mechanisms such as the NF-κB, C/EBPbd GATA4, mTOR, p38 MAPK, and cGAS-STING pathways and cytoplasmic chromatin fragments contribute to SASP activation (Salminen et al., 2012; Kang et al., 2015; Cuollo et al., 2020; Miller et al., 2021). Interactions of senescent cells with the microenvironment occur through cytoplasmic bridges, exosomes, NOTCH/JAG1 signaling, and ROS release, reflecting the complexity and importance of their impact on tissue function and health outcomes (Coppé et al., 2010; Biran et al., 2014; Takasugi et al., 2017).

## 5 Translational applications derived from epigenetics, inflammation, and cellular senescence in vascular disorders

### 5.1 Biomarkers of cellular senescence. Relevance in vascular aging

The study of cellular senescence has expanded considerably in recent years due to the discovery of numerous novel roles that this phenomenon plays in physiology and disease. The cell-cycle withdrawal is just one of the hallmarks of cellular senescence, which can be triggered by several stressors, such as telomere shortening, oxidative stress, DNA damage, and, oncogene activation (Muñoz-Espín and Serrano, 2014). Accordingly, cellular senescence has been reinterpreted as the process by which a dividing cell, in response to a stressful or damaging stimulus, enters a stable cell cycle arrest and usually secretes a complex mixture of substances that affect the surrounding tissue, while maintaining metabolic activity and resisting the signals of mitosis and apoptosis. Among other things, senescent cells have an enlarged and flattened shape, an expanded lysosomal compartment, and certain chromatin and epigenetic alterations (Beck et al., 2020). To accurately delineate cellular senescence in tissues and cell cultures, an optimal multifactorial approach requires meticulous organization and compilation of previously published methodologies and diverse approaches (González-Gualda et al., 2021). Senescence invites investigation of its involvement in diverse cellular pathophysiological processes, thus elucidating its four distinguishing features. Recently, single-cell time-lapse imaging revealed us that cell cycle withdrawal occurs gradually and not in a clear binary step (Ashraf et al., 2023). In addition, the intensity levels of senescence biomarkers appear to integrate the duration of earlier cell cycle withdrawal. The biomarkers related to epigenetic mechanisms and inflammation/SASP that regulate vascular senescence have been discussed in the previous sections. However, now we will take a look at the more general senescence biomarkers (Table 2).

**TABLE 2:** Biomarkers of cellular senescence and their respective functions.

Senescent trait	Biomarkers	Functional implications	Relevance in vascular senescence	References
Cell-cycle arrest	p53/p21^CIP1^	Inhibition of cell-cycle	OIS, DDR-induced senescence, ROS-induced senescence, replicative senescence	Chen (2016)
		The onset of cellular senescence		
	p16^INK4A^/pRB	Inhibition of cell-cycle	ROS-induced senescence, OIS, and replicative senescence	Grosse et al. (2020)
		Maintenance of cellular senescence		
	p38/CDKIs p15 and p27	Mitogenic activity	Role not clearly defined	Russo et al. (2020)
Metabolic dysregulation	SA-β-Gal	Lysosomal protein activity in senescent cells	Hydrolytic enzyme implication in β-galactosidase conversion to monosaccharide Evidence of increased lysosomal mass	Debacq-Chainiaux et al. (2009)
Macromolecular damage	Lipids of membrane	Altered levels	Increase in EPA, 7-HOCA, malonate, and 1-stearoylglycerophosphoinositol	James et al. (2015)
			Decrease in dihomo-linoleate, linoleate, and 10-heptadecenoate	
	Lipofuscin	Accumulation in senescent cells	Emerging as a hallmark of senescence. Role not clearly defined	Georgakopoulou et al. (2013), Evangelou and Gorgoulis (2017), Brickute et al. (2022)
	ROS	Cellular damage	ROS-induced senescence	Korovila et al. (2017)
	Telomeres	Telomere shortening	Replicative-induced senescence	
	Lamin B1	Loss of lamin B1	Disruption of the nuclear membrane and induction of senescence	Freund et al. (2012)
Epigenetic regulation	CpG Sites	Accumulation associated with histone variant H3.3	Links to DNA methylation alterations and chromatin accessibility	Cheng et al. (2017)
	Histone modifications	Gene expression regulation	Influences SASP, proliferation arrest, and gene enhancer activation	Salama et al. (2014)
	L1 retrotransposons	Activates cGAS-STING pathway	Induces type 1 interferon response, genomic instability, and SASP	Criscione et al. (2016)
	miRNAs	Targeted actions on various genes/proteins	Implicated in diverse vascular functions and pathogenic mechanisms	Menghini et al. (2014), Nikolajevic et al. (2022)
	AGO2	Complex formation with RB1	Facilitates CD2 and CDCA8 expression	Benhamed et al. (2012)
Inflammation (SASP)	IL-6	Implicated in hypertension	Strong correlation with hypertension; Lowered by angiotensin II-receptor blockade therapy	Passacquale et al. (2016), Stevenson et al. (2018)
	C-reactive protein	Triggered by inflammatory mediators	Aging risk factors and Inflammatory biomarker	Tang et al. (2017)
	GDF-15	Secreted by senescent endothelial cells	Impacts angiogenesis, apoptosis, and inflammation	Ha et al. (2019)

The cell-cycle arrest is one of the most distinctive features of cellular senescence and is indicated by a decrease in phosphorylated pRB and the protein markers p16, p21, and p53 (González-Gualda et al., 2021). As we have reviewed in the Introduction, the two main pathways that control this are the p53/p21^CIP1^ and p16^INK4A^/pRB axes. The former is observed in different pathways, such as oncogene-induced senescence (OIS), DNA damage response (DDR)-induced senescence, reactive oxygen species (ROS)-induced senescence, and replicative senescence. Following a DNA-damaging stimulus, p53 is activated through phosphorylation (p-p53), which then increases the temporary expression of the CDKI p21^CIP1^. After that, p21^CIP1^ suppresses CDK2-cyclin E, enabling pRB dephosphorylation and E2F sequestration to stop the cell cycle (Chen, 2016). On the other hand, the p16^INK4A^/pRB pathway is assumed to be more important in maintaining the quiescent state because it is normally engaged during ROS-induced senescence, OIS, and replicative senescence, meanwhile, it is not expressed during DDR-induced senescence. In this case, the protein p16^lnk4a^ directly inhibits the CDK4-Cyclin D complex upon activation of the *INK4a/ARF* genetic locus. This allows dephosphorylation and stability of the pRB-E2F complex, and consequently the inhibition of the cell cycle (Grosse et al., 2020). In both networks, pRB is the ultimate downstream target because, in its hypophosphorylated state, it binds E2F, a transcription factor that facilitates cell-cycle progression and entry into S-phase. Furthermore, the MAPK p38 and the CDKIs p15^INK4b^ and p27^KIP1^ can also be used as markers, however, their role in the senescent program is not as clear or widespread (Zhao et al., 2016; Russo et al., 2020).

To confirm the senescence phenotype or type of senescence, several markers associated with cell cycle arrest and SASP are usually analyzed along with other biomarkers. The presence of decreased expression of cyclins CCNA2, CCNE2, and LMNB1, a,s well as increased expression of a selection of SASP genes and the cyclin-dependent kinase inhibitors CDKN1A (p21WAF1/Cip1), CDKN2A (p16INK4A) and CDK2B (p15INK4B) need to be identified (Casella et al., 2019). These gene signatures are likely to be modified in the coming years due to the current lack of transcriptome datasets and the availability of further single-cell research to assess intrapopulation heterogeneity.

Secondly, there is a metabolic dysregulation. About this hallmark, the pathway that is most affected is the β-galactosidase (SA-β-Gal) (Cai et al., 2020; de Mera-Rodríguez et al., 2021). It is a hydrolase enzyme that catalyzes the conversion of β-galactosidase into monosaccharide in the lysosomes. The most frequent substrates for SA-β-Gal activity are galactose and 5-bromo-4-chloro-3-hydroxyindole-1. This enzyme assay, which uses X-Gal as a chromogenic substrate, tracks the elevated expression and activity of this lysosomal protein in senescent cells and provides evidence of an increase in lysosomal mass (Debacq-Chainiaux et al., 2009; Valieva et al., 2022). The senescent cells differ in several ways from normal in terms of mitochondrial dynamics, function, and appearance. Senescent cells have fewer functioning mitochondria, which exhibit reduced membrane potential higher proton leakage, decreased rates of fusion and fission, increased bulk, and a greater quantity of metabolites related to the tricarboxylic acid cycle (TCA cycle) (Kaplon et al., 2013). Also, it can be brought on by damaging elements of mitochondrial biology, like the electron transport chain (ETC.), complex I assembly, and/or mitochondrial sirtuins (Correia-Melo et al., 2016). On the other hand, senescent cells frequently create higher levels of ROS, which can lead to telomere shorting DDR activation in addition to the protein and lipid degradation covered in earlier sections. SASP regulation is also associated with mitochondrial dysfunction during senescence (Martini and Passos, 2023). Senescent cells appear to reduce the SASP by mitophagy, or mitochondrial clearing. Even when cells do not express important proinflammatory SASP factors like IL-6 and IL-8, senescence can still be induced by genetic or pharmacological suppression of the ECT (Wiley et al., 2016; Wiley and Campisi, 2021).

The macromolecular damage in cellular senescence comprises lipids, proteins, and nucleic acids affecting the cellular structures. Regarding the plasma membrane, both its integrity and signal transduction depend on the lipid content. Changes in lipid profiles result from altered lipid metabolism, a characteristic of several age-related illnesses (Ademowo et al., 2017). Senescent fibroblasts exhibit an increase in fatty acids, their precursors, and phospholipid catabolites, such as eicosapentaenoic (EPA), 1-stear-oylglycerophosphoinositol, malonate, and 7-alpha-hydroxy-3-oxo-4-cholestenoate (7-HOCA), whereas dihomo-linoleate, linoleate, and 10-heptadecenoate decrease (James et al., 2015). ROS is a well-known cause of protein damage because it oxidizes cysteine and methionine residues, changing the way proteins fold and function (Korovila et al., 2017). Threonine, proline, lysine, and arginine residues are carbonylated. Protein carbonylation makes hydrophobic surfaces visible, which causes unfolding and aggregation. Furthermore, carbonyl residues can contribute to protein aggregation by reacting with amino groups to create Schiff bases. After further cross-linking with lipids and carbohydrates, lipofuscin insoluble aggregate is created (Evangelou et al., 2017). It accumulates as a byproduct of senescence in senescent cells and ought to be regarded as a new “hallmark” of senescence (Georgakopoulou et al., 2013). It is commonly recognized that the Sudan-Black-B (SBB) histochemical stain will only react with lipofuscin, which is a mixture of oxidized proteins, lipids, and metals (Evangelou and Gorgoulis, 2017). It has also been observed to build up in senescent cells. It is demonstrated that the use of SBB staining yields very specific results when it comes to senescent cell imaging (Brickute et al., 2022). This offers special benefits for understanding the physiological mechanism and the pathophysiology of different age-related diseases as well as for predicting how well treatment approaches will work.

### 5.2 Senotherapy

Given the great importance that scientific evidence has given to senescence in the development of age-related pathological processes, the field of senotherapy has experienced exponential growth throughout the last decade. At first, two big groups of senotherapeutic compounds were identified: senolytics and senomorphics. Senolytics selectively induce cell death of senescent cells, mostly by targeting elements of anti-apoptotic and pro-survival pathways of the cell (tyrosine kinases, Bcl-2 protein family, MDM2, FOXO4, HDACs) (Table 3), while senomorphics modulate the SASP, responsible of many of the deleterious effects associated to senescence (by targeting telomerase, sirtuins, mTOR, NF-kB, ATM JAK/STAT, p38/MAPK) (Table 4) (Ya and Bayraktutan, 2023).

**TABLE 3:** Senolytics: effects in vascular cells and clinical trials for vascular diseases. BPTES [bis-2-(5-phenyl- acetamido-1,3,4-thiadiazol-2-yl) ethyl sulfide].

Compound	Mechanism of action	Outcomes			References
		*In vitro*	*In vivo*	Clinical trials (study phase, identifier)	
Dasatinib + quercetin (D + Q)	Tyrosine kinase inhibition (Dasatinib) PI3K and Bcl-2 family members inhibition (Quercetin)	-D + Q afforded selective killing of both senescent preadipocytes and ECs	-Improved cardiac function and carotid vascular reactivity in aged mice	Epigenetic Aging (Phase 2, NCT04946383) Chronic Kidney Disease (Phase 2, NCT02848131) Alzheimer’s disease (Phase 2, NCT04063124) (Phase 2, NCT04785300) (Phase 2, NCT05422885) Idiopathic pulmonary fibrosis (Phase 1, NCT02874989) Accelerated aging in mental disorders (Phase 2, NCT05838560)	Zhu et al. (2015), Parvizi et al. (2021), Lin et al. (2023)
		-Administration of D + Q could selectively clear senescent cells and preserve the caveolar CaV3.2-RyR axis in aging VSMCs	-Attenuation of the enlargement of the abdominal aorta induced by angiotensin II in aged mice		
ABT-263 (Navitoclax)	Bcl-2/Bcl-XL family member inhibitor	-Senolytic activity in senescent HUVECs	Reduction of atherosclerosis in mice	-	Childs et al. (2016), Zhu et al. (2016), Garrido et al. (2022)
		-Selective cell death of murine senescent VSMCs			
UBX-1967	Inhibition of BCL-xL	Elimination of senescent cells and suppression of neovascularization while enhancing vascular repair in a model of retinopathy	The same results in the mouse model of retinopathy	-	Crespo-Garcia et al. (2021)
UBX-1325 (Fuselutoclax)	Inhibition of BCL-xL	Selective elimination of senescent cells	Inhibited retinal neovascularization and reduced vascular leakage	Diabetic macular edema (Phase 2, NCT06011798) (Phase 2, NCT04857996) Neovascular age-related macular degeneration (Phase 2, NCT05275205)	Hassan and Bhatwadekar (2022)
STA-9090 (Ganetespib)	Hsp90 inhibitor	Senolytic activity in senescent HUVECs	-	-	Fuhrmann-Stroissnigg et al. (2017a)
FOXO4-DRI	Disruption of FOXO4-p53 interaction	Selective induction of cell death of senescent fibroblasts	-	-	Baar et al. (2017)
P5091, P22077	USP7 inhibitors	Induction of apoptosis in senescent fibroblast cell lines and senescent HUVECs	-	-	He et al. (2020)
Fisetin	Diverse signalling pathways, including BCL-2, PI3K/AKT, p53, NF-kB	Induction of apoptosis in senescent, but not in proliferating HUVECs	Reduction of senescence markers in multiple tissues, restoration of tissue homeostasis, reduction of age-related pathology, and extension of lifespan of progeroid and old mice	Osteoarthritis (Phase 2, NCT04210986) Inflammation and frailty (Phase 2, NCT03675724) (Phase 2, NCT03430037) COVID-19 Inflammation (Phase 2, NCT04476953), (Phase 2, NCT04771611) Improvement of vascular function in older adults (Phase 2, NCT06133634)	Fuhrmann-Stroissnigg et al. (2017b), Yousefzadeh et al. (2018)
Piperlongumine and analogues	Induction of proteasomal degradation of OXR1 and increased production of ROS	Induction of cell death in senescent fibroblast cells	-	-	Liu et al. (2018)
Cardiac glycosides	Targeting autophagy or through inhibition of Na+/K + -ATPase	-Proscillaridin A, Ouabain, and Digoxin showed specific senolytic activity in human BJ fibroblasts induced to senescence by Bleomycin treatment	-	-	Triana-Martínez et al. (2019), L’Hôte et al. (2021)
		-Ouabain triggers senolysis in a cell line of BRAF-senescent human fibroblasts			
BPTES	Glutaminase 1 inhibitor	BPTES exerted senolytic activity in senescent fibroblast cell lines	Reduction of atherosclerosis of thoracic aorta and plaque lesions and senescence markers in abdominal aorta in mice	-	Johmura et al. (2021)

**TABLE 4:** Senomorphics: effects in vascular cells and clinical trials for vascular diseases. COPD: chronic obstructive pulmonary disease.

Compound	Mechanism of action	Outcomes			References
		*In vitro*	*In vivo*	Clinical trials (study phase, identifier)	
Rapamycin/sirolimus	mTORC1 inhibitor	mTOR inhibition by low-dose rapamycin prevented cell senescence and inhibited the SASP in cultured cells derived from patients with COPD	Significant reduction in atherosclerotic lesions area and improved neurovascular measures in mice	Immune, Cognitive, and Functional Consequences of mTOR inhibition in the elderly (including assessment of cardiac function) (Phase 2, NCT04742777) (Phase 2, NCT02874924) Angiofibromas in Tuberous sclerosis (Phase 2, NCT01526356) Alzheimer’s and Cognitive Health (Phase 2, NCT04629495)	Houssaini et al. (2018), Jahrling et al. (2018)
Metformin	Numerous cellular targets. Some related to SASP inhibition are: IKK inhibition, upregulation of Nrf2-mediated glutathione peroxidase 7 (GPx7), or downregulation of the STAT3 pathway	Metformin decreased the secretion of SASP factors and adhesion molecules, as well as LPS-triggered hyper-inflammation, in doxorubicin-induced senescent ECs	Metformin reduced pro-inflammatory markers such as CCL2 and also improved oxidative stress, nitric oxide bioavailability, and endothelial dysfunction in general in a murine model of non-obese type 2 diabetes	Treatment of frailty in obese seniors (Phase 3, NCT04221750) Peripheral Arterial Calcification in Type 1 Diabetes (Phase 3, NCT04583462) Peripheral artery disease (Phase 3, NCT05132439)	Sena et al. (2011), Abdelgawad et al. (2023)
Aspirin	Unknown	Inhibition of astrocyte-driven inflammation by targeting cGAS in a brain organoid model of ataxia-telangiectasia	Normalization of vascular remodeling of renal and carotid arteries, and reversion of the increment in systolic blood pressure on a model of metabolic syndrome	Cerebral Small Vessel Disease (Phase 4, NCT01932203) Primary prevention of cardiovascular events in the elderly (Phase 4, NCT00225849) Secondary prevention of CVD in the elderly (Phase 3, NCT02596126)	Renna et al. (2009), Aguado et al. (2021)
Statins	HMG-CoA reductase inhibitor	Atorvastatin, pravastatin, and pitavastatin inhibited senescence in a cell line of senescent HUVECs, by targeting the Akt pathway	-	-	Ota et al. (2010)
Ruxolitinib	JAK 1/2 inhibitor	Reduction of the levels of some key SASP components in senescent HUVECs, including IL-6, IL-8, and MCP-1	Reduction of systemic inflammation in aged mice	-	Xu et al. (2015)
Oridonin	p38 and NF-κB inhibitor	Inhibition of the secretion of IL-8 and IL-6 in a cell line of human senescent fibroblasts	-	-	Yasuda et al. (2022)
Curcumin	Unknown, probably by downregulation of Nrf2 and NF-kB pathways	Senescent HUVECs induced to exert a pro-inflammatory response significantly reduced the expression of pro-inflammatory markers such as MCP-1	-	Chronic Kidney Disease (recruiting, NCT04413266)	Matacchione et al. (2022)
Resveratrol	SIRT-1 and AMPK activator	Prevention of the onset of senescence in cultured endothelial progenitor cells, and also an increase of the proliferation and migration of these cells	Protective effects against arterial aging by reducing the aorta media thickness, inflammation, fibrosis, and oxidative stress and reduction of the number of senescent VSMCs in mice	Cardiovascular Health in the Elderly (Phase 2, NCT01842399)	Xia et al. (2008), Kim et al. (2018)
	Inhibitor of the NF-kB pathway			Vascular system and lipid metabolism disorders in Women and Men Aged 55–65 Years (NCT01668836)	
Apigenin	IRAK1/IkBa/NF-kB inhibition	Reduction of the levels of SASP factors (IL-1α, IL-1β, IL-6, IL-8, GM-CSF, CXCL1, MCP-2 and MMP-3) detected in a cell line of senescent fibroblasts	The same outcomes in the kidneys of aged rats	Improvement of Organ Function by Apigenin in Elderly Patients with Sepsis (Phase 2, NCT05999682)	Lim et al. (2015)

Over time, new senotherapeutic approaches emerged, addressing some of the limitations that senolytics or senomorphics compounds presented. For example, the development of galactose-modified senolytic prodrugs allowed guiding the action of these molecules more specifically toward senescent cells (Zhang et al., 2022). It consists of conjugating the senolytic compound with a galactose moiety, which would be cleaved by the senescent cells, given the increased β-galactosidase activity displayed by these cells; this way, the active compound would be released, selectively killing the senescent cell. This strategy has been already tested with some senolytics such as Navitoclax (González-Gualda et al., 2020). Other approaches involve the use of small peptides targeting surface molecules of these cells, like CD47, implicated in the inhibition of phagocytosis of these cells by the immune system, and therefore restoring the clearance of senescent cells (Jatal et al., 2022). Also worth mentioning is the development of senescence immunotherapy. One example of this is the use of functionalized nanoparticles with antibodies to CD9, a surface cell protein upregulated in endothelial cell senescence, to deliver rosuvastatin, a senomorphic agent, to these cells (Pham et al., 2021). Lastly, it is important to highlight the potential of therapeutic strategies that attempt to induce senescence, particularly interesting to enhance the effectiveness of other treatments, such as anti-cancer therapy. Certain studies provide evidence that inducing senescence of endothelial cells in a preclinical model leads to the production of pro-inflammatory and angiogenic factors by these cells, stimulating the immune response and sensitizing unresponsive tumors to immunotherapy (Ruscetti et al., 2020).

However, it is important to mention that senotherapy has still numerous challenges to confront. Firstly, senescent cells are heterogeneous between species, and cell types, even within the same cell type, and rely on different senescence-mediating pathways from 1 cell to another (Zhang et al., 2023). Secondly, not all senescence-associated metabolic alterations are cell-specific, which may lead to undesired off-target events of senotherapeutics, like rapamycin, which apart from its senomorphic properties, has been shown to accelerate the progression of cultured endothelial progenitor cells to a senescent state or cause impaired vasorelaxation by dysregulation of superoxide and NO production (Imanishi et al., 2006; Jabs et al., 2008). Added to this is the important role that senescent cells play in processes such as tissue renewal, wound healing, tumor suppression, and others, which is why senotherapeutics must allow beneficial senescent cell populations to persist (Zhang et al., 2023). Indiscriminate elimination of senescent cells in some situations and conditions may have harmful effects, as described in this study where elimination of senescent pulmonary endothelial cells by FOXO4-DRI worsened pulmonary hemodynamics in a murine model of pulmonary hypertension (Born et al., 2023).

Nevertheless, a multitude of pathologies can potentially be alleviated by the use of senotherapeutics, as shown by the favorable results of many current clinical trials. Many of these are vascular diseases such as fibrotic conditions, atherosclerotic disease, pulmonary arterial hypertension, or peripheral artery disease, among others (Suda et al., 2023). Table 3 and Table 4 list some of the most studied senolytic and senomorphic compounds in pathologies related to vascular alterations, as well as their mechanisms of action, main outcomes of the studies performed, and clinical trials carried out.

## 6 Conclusion

Cellular senescence is a state in which the cell leaves the cell cycle in response to damage signals or developmental cues to prevent tumor proliferation and promote cell survival. The senescent cell remains viable despite macromolecular damage and dysregulated metabolism and acquires a SASP. Cellular senescence is a hallmark of aging because its accumulation in tissues is detrimental to tissue and organismal homeostasis. Some studies that selectively eliminate these cells in animal models manage to prolong their lifespan. In this regard, we have reviewed the impact of cellular senescence on the vascular wall, termed vascular senescence, which appears to be one of the most important drivers of vascular aging. The best characterized and studied cell types that become senescent and contribute to vascular dysfunction are endothelial cells, vascular smooth muscle cells, adventitial fibroblasts, and immune cells. In general, they produce oxidative stress and secretion of proinflammatory factors that damage the vascular wall. This has been found in age-related vascular diseases such as atherosclerosis, peripheral artery disease, hypertension, chronic venous disease, or venous ulcers.

Multiple epigenetic mechanisms are activated during the senescence state, including the expression of microRNAs, chromatin remodeling, DNA methylation, histone modification, and loss of nuclear integrity. This implies upregulation in the expression of inflammation-related genes, mainly those that promote SASP, such as proinflammatory cytokines, chemokines, chemokine receptors, and endothelial adhesion molecules. Of interest is the investigation of specific biomarkers of vascular senescence in different blood vessel types and cell types. Identifying unique signatures in different cell types and blood vessels can improve disease detection and response to treatments of vascular diseases. Moreover, exploring the inflammatory response activated during vascular senescence may reveal new therapeutic targets to treat age-related vascular diseases. In addition, understanding this complex process allows us the application of a promising therapeutic approach, senotherapy. Further research is needed to better characterize the different senotherapeutic options, senolytics and senomorphics, and to translate the results of research in preclinical models and clinical trials into clinical practice, improving diagnostic accuracy, prognostic ability, and therapeutic interventions for vascular senescence-related conditions.
